# A Review of the Correlation Between Epidermal Growth Factor Receptor Mutation Status and ^18^F-FDG Metabolic Activity in Non-Small Cell Lung Cancer

**DOI:** 10.3389/fonc.2022.780186

**Published:** 2022-04-20

**Authors:** Maoqing Jiang, Xiaohui Zhang, Yan Chen, Ping Chen, Xiuyu Guo, Lijuan Ma, Qiaoling Gao, Weiqi Mei, Jingfeng Zhang, Jianjun Zheng

**Affiliations:** ^1^ Department of PET/CT Center, Hwa Mei Hospital, University of Chinese Academy of Sciences, Ningbo, China; ^2^ Ningbo Institute of Life and Health Industry, University of Chinese Academy of Sciences, Ningbo, China; ^3^ Department of Nuclear Medicine, Hwa Mei Hospital, University of Chinese Academy of Sciences, Ningbo, China; ^4^ Department of Physical Examination Center, Ningbo First Hospital, Ningbo, China; ^5^ Department of Nephrology, Hwa Mei Hospital, University of Chinese Academy of Sciences, Ningbo, China; ^6^ Department of Education, Hwa Mei Hospital, University of Chinese Academy of Sciences, Ningbo, China

**Keywords:** non-small cell lung cancer, epidermal growth factor receptor, tyrosine kinase inhibitors, positron emission tomography, ^18^F-FDG

## Abstract

PET/CT with ^18^F-2-fluoro-2-deoxyglucose (^18^F-FDG) has been proposed as a promising modality for diagnosing and monitoring treatment response and evaluating prognosis for patients with non-small cell lung cancer (NSCLC). The status of epidermal growth factor receptor (EGFR) mutation is a critical signal for the treatment strategies of patients with NSCLC. Higher response rates and prolonged progression-free survival could be obtained in patients with NSCLC harboring EGFR mutations treated with tyrosine kinase inhibitors (TKIs) when compared with traditional cytotoxic chemotherapy. However, patients with EGFR mutation treated with TKIs inevitably develop drug resistance, so predicting the duration of resistance is of great importance for selecting individual treatment strategies. Several semiquantitative metabolic parameters, e.g., maximum standard uptake value (SUV_max_), metabolic tumor volume (MTV), and total lesion glycolysis (TLG), measured by PET/CT to reflect ^18^F-FDG metabolic activity, have been demonstrated to be powerful in predicting the status of EGFR mutation, monitoring treatment response of TKIs, and assessing the outcome of patients with NSCLC. In this review, we summarize the biological and clinical correlations between EGFR mutation status and ^18^F-FDG metabolic activity in NSCLC. The metabolic activity of ^18^F-FDG, as an extrinsic manifestation of NSCLC, could reflect the mutation status of intrinsic factor EGFR. Both of them play a critical role in guiding the implementation of treatment modalities and evaluating therapy efficacy and outcome for patients with NSCLC.

## Introduction

In 2020, it was estimated that there were approximately 228,820 newly diagnosed lung cancer cases and 135,720 deaths from lung cancer in the United States (1). Non-small cell lung cancer (NSCLC), a major phenotype of lung cancer, accounting for about 80%–85%, is one of the leading causes of cancer-related deaths worldwide despite improvements in diagnostic and therapeutic modalities (1, 2). Epidermal growth factor receptor (EGFR) mutations were found in about 35% of patients with NSCLC in East Asia and 10%–15% in the United States (3, 4). In addition, EGFR mutations were demonstrated to be significantly associated with adenocarcinoma, never smoking, and the female gender (5). Patients with EGFR mutations treated with tyrosine kinase inhibitors (TKIs) were linked to a higher response rate and longer progression-free survival (PFS) than those treated with conventional cytotoxic chemotherapy (6, 7). Eventually, however, resistance to TKIs inevitably occurred with a median PFS of 9 to 13 months (7–9). In this regard, accurate prediction of EGFR mutations and monitoring of TKI response rates and drug resistance will be of great value for clinicians to perform individual treatment strategies.

PET/CT with ^18^F-2-fluoro-2-deoxyglucose (^18^F-FDG) has been widely used for pretreatment staging and restaging, monitoring treatment response, and evaluating prognosis for patients with NSCLC (10–14). Several semiquantitative metabolic parameters, e.g., maximal standard uptake value (SUV_max_), total lesion glycolysis (TLG), and metabolic tumor volume (MTV), have been demonstrated to be promising PET/CT indices to reflect the metabolic activity and/or tumor burden (15, 16). SUV_max_, a parameter representing the maximum uptake value of ^18^F-FDG in a single-pixel adjusted for lean body mass, has been widely used as a marker for glucose metabolic activity, but it cannot clearly reflect tumor burden. TLG, a quantitative volume-based metabolic PET parameter, has been recognized as a promising index for its advantages to reflect the metabolic activity and tumor burden. Higher SUV_max_, TLG, or MTV on ^18^F-FDG PET/CT scan usually revealed a short PFS or overall survival (OS) for patients with NSCLC (17–19). Consequently, a certain cross and overlap may have occurred between the roles of ^18^F-FDG PET/CT and EGFR in evaluating the efficacy and outcome of NSCLC patients.

Over the past two decades, a great number of studies have attempted to elucidate the relationship between the status of EGFR mutation and the metabolic activity of ^18^F-FDG in NSCLC (20–23). Obviously, EGFR mutation status represents an intrinsic factor of NSCLC, while ^18^F-FDG metabolic activity is an extrinsic manifestation of NSCLC. There is a close association between EGFR mutation status and ^18^F-FDG metabolic activity in NSCLC, but the relationship between them needs to be further clarified due to contradictory reports (24–26). A large sample study including 849 patients with NSCLC showed that low SUV_max_ of the primary tumor, lymph node, and distant metastasis were associated significantly with EGFR mutations (24), whereas another study presented opposite results that high SUV_max_ (≥6.0) of the primary tumor was more likely to have EGFR mutations in NSCLC (25). In addition, no significant difference in ^18^F-FDG uptake between mutant EGFR and wild-type EGFR was also observed in NSCLC patients (26). Accordingly, in this work, we aimed to comprehensively review the biological and clinical correlations between EGFR mutation status and ^18^F-FDG metabolic activity in NSCLC.

## Biological Correlation Between Epidermal Growth Factor Receptor Mutation Status and ^18^F-FDG Metabolic Activity in Non-Small Cell Lung Cancer

**Figure 1:**
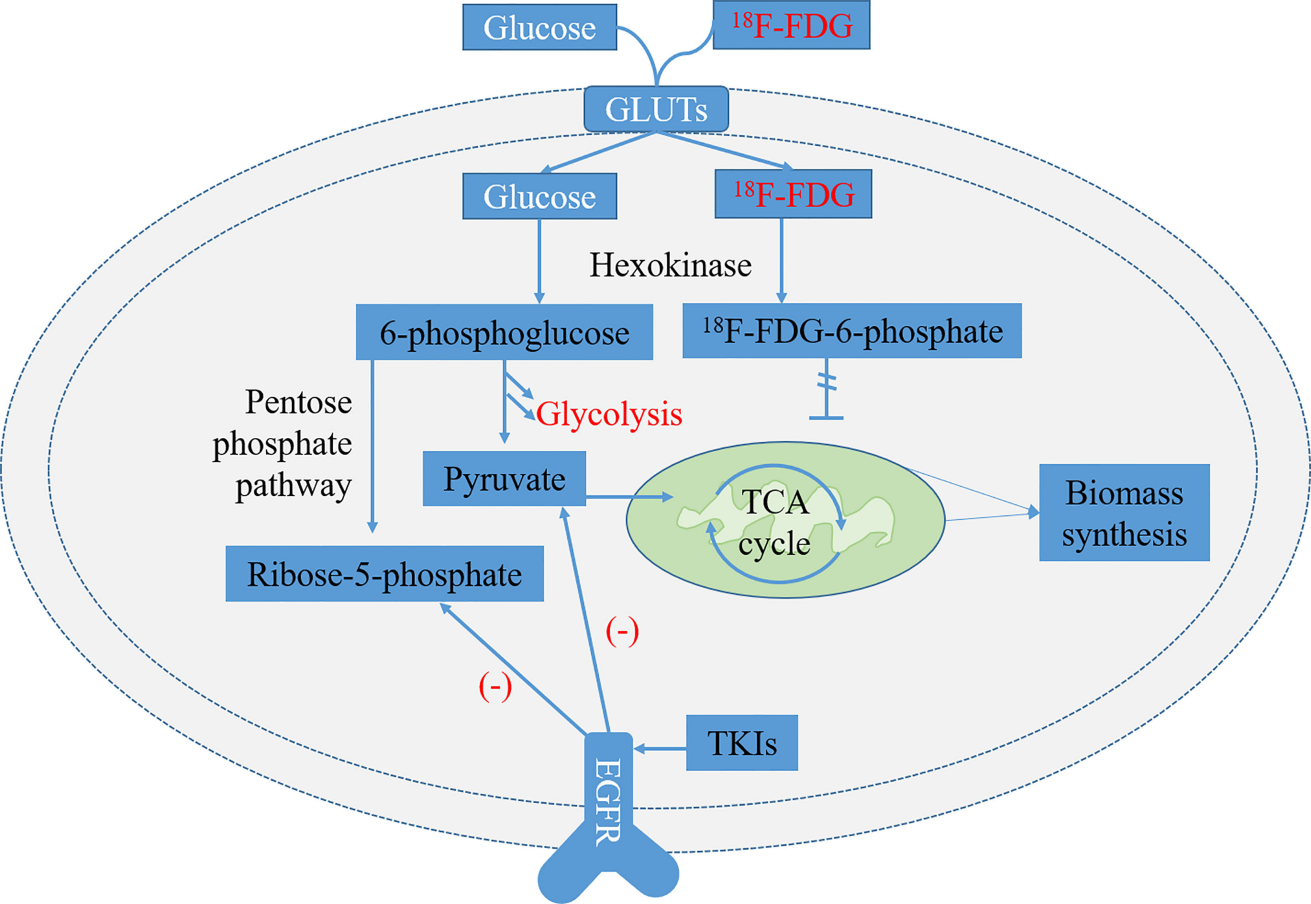
Glycolysis pathways of ^18^F-FDG and normal glucose and related metabolic pathways regulated by epidermal growth factor receptor (EGFR) signaling in EGFR-mutated non-small cell lung cancer (NSCLC). ^18^F-FDG is transported into cells by glucose transporters (GLUTs) and phosphorylated to ^18^F-FDG-6-phosphate by hexokinase (HK). It is trapped inside cells because ^18^F-FDG-6-phosphate is not a substrate of glycolysis or pentose phosphate pathway (PPP) and is unable to diffuse outside cells. The metabolites of pyruvate and ribose-5-phosphate in the glycolysis decreased significantly after treatment of lung adenocarcinoma cells with EGFR tyrosine kinase inhibitors (TKIs) (33).

Tumor cells utilize a variety of metabolic pathways, especially glucose, to meet the requirements of bioenergy and biosynthesis for growth and proliferation (27, 28). Oncogenic mutations are the driving force of high energetic metabolism that can be maintained persistently in cancer cells (29). In addition, glucose metabolism preferentially tends to aerobic glycolysis rather than mitochondrial oxidative phosphorylation, which is known as the Warburg effect (27). It has been reported that many oncogenic signaling pathways in cancer cells, particularly EGFR aberrant signaling, lead to the metabolic switch from mitochondrial oxidative phosphorylation to aerobic glycolysis (30, 31). Recently, EGFR has been identified as a driver of oncogenes in NSCLC, because the mutation of activating EGFR kinase domain enhances the activity of EGFR tyrosine kinase, leading to continuous activation of the downstream signal pathway, and then drives tumorigenesis and tumor progression (32). Targeted EGFR mutation therapies, such as EGFR-TKIs, including erlotinib and gefitinib, have shown to be highly effective in inhibiting glucose consumption in both *in vitro* and *in vivo* models of NSCLC (**Figure 1**) (33, 34).

^18^F-FDG, a glucose analog, is transported into cells by glucose transporters (GLUTs) and phosphorylated to ^18^F-FDG-6-phosphate by hexokinase (HK). It is trapped inside cells and dephosphorylated slowly because ^18^F-FDG-6-phosphate is not a substrate of glycolysis or pentose phosphate pathway (PPP) and is unable to diffuse outside cells (**Figure 1**) (35). Now, it has been widely used as a small molecule radiotracer for PET/CT imaging and has been applied extensively as a tracer to reflect glucose metabolic activity in diagnosing and evaluating treatment response of various malignant tumors, including NSCLC (36, 37). The overexpression of GLUT1 and HK-I is highly associated with the increased uptake of ^18^F-FDG in NSCLC, showing that the uptake of ^18^F-FDG seems to be regulated by glucose metabolism (38–40).

Several mutated oncogenes have been demonstrated to be associated with metabolic signaling pathways that affect tumor cell metabolism (41). In EGFR-mutated adenocarcinoma cells, lactate production, glucose-induced extracellular acidification rate, and glucose consumption were significantly decreased after treatment with TKIs, showing that EGFR signaling played a major role in aerobic glycolysis (33). In gefitinib-sensitive NSCLC cell lines with EGFR mutations, the uptake of ^18^F-FDG was also decreased significantly as early as 2 h after treatment, whereas no measurable changes in ^18^F-FDG uptake were observed in gefitinib-resistant cells, representing treatment response of gefitinib that could be closely reflected by glucose metabolic activity (34). Accordingly, to a certain extent, the metabolic activity of ^18^F-FDG in NSCLC cell lines is correlated with or may reflect the mutations of EGFR.

## Clinical Correlation Between Epidermal Growth Factor Receptor Mutation Status and ^18^F-FDG Metabolic Activity in Non-Small Cell Lung Cancer

### Predicting Epidermal Growth Factor Receptor Mutation Status With ^18^F-FDG PET/CT

A large number of studies reported that compared with traditional cytotoxic chemotherapy, NSCLC patients with EGFR mutation treated with TKIs had a higher response rate and prolonged PFS (6, 7). The presence of EGFR gene mutations in lung adenocarcinoma is a powerful predictor of better prognosis after gefitinib therapy (9). Accordingly, the status of EGFR mutations plays a critical role in selecting suitable treatment modalities for patients with NSCLC. However, in clinical practice, the status of EGFR mutation is usually determined by tissue-based analysis (42), which has a number of limitations, e.g., i) sampling bias due to tumor heterogeneous, ii) associated complications owing to invasive biopsies, iii) not rapid and expensive, and iv) failing to get reliable results due to the low quantity or quality of the tissue samples (43). In addition, the mutation status of EGFR may be changed in the course of chemotherapy or targeted therapy (44). Therefore, a non-invasive method is urgently needed to monitor EGFR mutation status in NSCLC.

PET/CT scan with ^18^F-FDG, a non-invasive and functional imaging method, has a powerful ability to predict the mutation status of EGFR in NSCLC (45–47). SUV_max_ is the most widely used index of ^18^F-FDG PET/CT in predicting EGFR mutations (24). Patients with NSCLC harboring EGFR mutations usually showed lower SUV_max_ than those with wild-type EGFR (**Table 1**) (21, 24, 48, 49). Normally, SUV_max_ was calculated only from the primary lesions of NSCLC, whereas the distant metastasis and/or metastatic lymph nodes were also monitored in some studies (24, 50). Low SUV_max_ of the distant metastasis was beneficial to the existence of EGFR mutations in advanced lung adenocarcinoma (50). Different cutoff values of SUV_max_ (range, 7.0–9.91) were determined to obtain a relatively high receiver operating characteristic (ROC) curve area (range, 0.557–0.75) (20, 24, 50). In addition to SUV_max_, MTV was also used as a parameter to predict EGFR mutations in NSCLC. Patients with NSCLC harboring EGFR mutation had lower MTV than those with wild-type EGFR (57). Interestingly, the serum carcinoembryonic antigen (CEA) can increase during all adenocarcinomas not only in those EGFR mutated but also in wild type (58). The combination of serum CEA and SUV_max_ was also performed to predict EGFR mutations in patients with NSCLC, which demonstrated to have a moderate diagnostic accuracy (25, 51).

**Table 1:** The clinical and pathological features, glucose metabolic activity, and EGFR mutation status in NSCLC of previous studies.

Studies	No. of patients		Stage (n)		Histopathology (n)			Lesions measured			Metabolic parameters			EGFR status (n)		Metabolic parameters favor EGFR mutation in NSCLC
	Male	Female	I–II	III–IV	ADC	SCC	Other	PT	LN	MT	SUV_max_	MTV	TLG	Mutant	Wild type	
Lv et al. (24)	468	340	191	617	731	58	19	√	√	√	√	–	–	371	437	Low SUV_max_ in PT
Mak et al. (21)	39	61	40	60	55	2	43	√	–	√	√	–	–	24	76	Low SUV_max_ in PT
Cho et al. (48)	33	28	26	35	57	2	2	√	–	–	√	–	√	30	31	Low SUV_max_ in PT
Gao et al. (49)	87	80	8	159	162	5	0	√	√	–	√	–	–	73	94	Low SUV_max_ in PT and LN
Lee et al. (50)	33	38	0	71	71	–	–	√	√	√	√	–	–	48	23	Low SUV_max_ in MT
Na et al. (20)	68	32	57	43	53	40	7	√	–	–	√	–	–	21	79	Low SUV_max_ in PT
Gu et al. (51)	132	78	58	152	161	34	15	√	–	–	√	–	–	70	140	Low SUV_max_ (<9.0) in PT
Ko et al. (25)	57	75	49	83	132	–	–	√	–	–	√	–	–	69	63	High SUV_max_ (>6.0) in PT
Wang et al. (52)	189	122	40	271	233	44	34	√	–	√	√	–	–	128	183	High SUV_max_ (>11.2) in PT
Kanmaz et al. (53)	151	67	18	200	218	–	–	NS	NS	NS	√	–	–	63	155	High SUV_max_
Huang et al. (54)	33	44	0	77	77	–	–	√	–	–	√	–	–	49	28	Higher SUV_max_ in PT
Chung et al. (15)	63	43	19	87	106	–	–	√	√	√	√	√	√	42	64	No correlation
Choi et al. (55)	99	64	0	163	130	27	6	√	–	–	√	–	–	57	106	No significant difference of SUV_max_ in PT
Caicedo et al. (26)	62	40	0	102	88	6	8	NS	NS	NS	√	–	–	22	80	No significant difference of SUV_max_
Lee et al. (56)	148	58	22	184	135	71	–	√	–	–	√	–	–	47	159	No significant difference

ADC, adenocarcinoma; SCC, squamous cell carcinoma; PT, primary tumor; LN, lymph nodes; MT, metastatic; MTV, metabolic tumor volume; TLG, total lesion glycolysis; EGFR, epidermal growth factor receptor; NSCLC, non-small cell lung cancer; NS, not specified; “-”, not done.

However, opposite results could be observed that the metabolic activity of ^18^F-FDG (e.g., SUV_max_) in NSCLC EGFR-mutant patients was significantly higher than that of wild-type patients (25, 52–54). The expression status of EGFR protein was also evaluated, and higher SUV_max_ was positively correlated with EGFR overexpression (59, 60). Furthermore, no significant difference in ^18^F-FDG uptake was observed between EGFR mutant and wild-type NSCLC patients in previous reports (**Table 1**) (26, 55, 56). Several reasons could lead to these conflicting results. First, the number of patients included in the studies varied widely, as low as only 61 patients and as high as up to 808 patients (24, 48, 57). Second, the rate of EGFR mutations varied greatly among NSCLC patients, from 21% to 68% (20, 50). Third, the proportion of histopathological subtypes of NSCLC (adenocarcinoma and squamous cell carcinoma) varied significantly, as EGFR mutations are difficult to detect in squamous cell carcinoma patients who smoke, while EGFR mutations are more common in adenocarcinoma (20, 21, 61). Fourth, the clinical stage (I–II vs. advanced stage) of patients with NSCLC was significantly different (25, 26). More importantly, multiple objective reasons, e.g., different PET/CT scanners, the plasma glucose level before PET/CT scan, fasting time, and region of interest parameters might result in contradictory results. Therefore, many novel techniques of PET/CT are performed to investigate the predictive efficacy of EGFR mutations in NSCLC.

Radiomics, an advanced mathematical model for quantifying the spatial relationships among image voxels, has become a growing research field in which a great number of imaging features are investigated in order to choose the most significantly relevant features with clinical, pathological, molecular, and genetic features, so as to improve the accuracy of diagnosis, prognosis, and curative effect evaluation (62, 63). Accordingly, the role of ^18^F-FDG PET/CT radiomics in predicting EGFR mutation status for patients with NSCLC has been evaluated (47, 64–67). The area under the ROC curve (AUC) was usually in the range of 0.57 to 0.86 when based on the radiomics features of PET/CT, whereas the performance would get a significantly higher efficacy when combined with clinical features and/or conventional PET/CT parameters, such as SUV_max_, SUVmean, MTV, and TLG (47, 67, 68). In addition, four exons (18–21) of EGFR mutations have been observed in NSCLC patients (69), in which approximately 90% are exon 21 L858R substitutions and exon 19 deletions (70). Recently, research showed that two sets of prognostic radiomics features of ^18^F-FDG PET/CT could distinguish EGFR exon 19 deletions from EGFR exon 21 L858R missense, with an AUC of 0.87 in predicting EGFR mutation status (46).

In short, detection of EGFR mutation status in NSCLC plays a major role in the daily management of individual patients, especially in the selection of TKI targeted therapy. ^18^F-FDG PET/CT has been demonstrated to have a powerful efficacy to predict the EGFR mutation status in patients with NSCLC, not only based on conventional PET/CT parameters (e.g., SUV_max_, MTV, and TLG) but also based on radiomics of PET/CT. The combination of clinical features, laboratory results, conventional PET/CT parameters, and PET/CT radiomics would provide higher accuracy in predicting EGFR mutation status. However, there are still many contradictory reports, so ^18^F-FDG PET/CT should be used with caution when predicting EGFR mutations in patients with NSCLC. More prospective cohort studies are needed to further verify the role of ^18^F-FDG PET/CT in predicting EGFR mutations.

### Evaluating Treatment Response for Patients With Non-Small Cell Lung Cancer

Most patients with NSCLC develop late in the course of the disease, which is inoperable (12, 71). The standard treatment modality for those patients remains systematic chemotherapy (72). However, since not all patients with NSCLC respond well to chemotherapy and the treatment is toxic, it is important to identify those patients who are less or most likely to benefit from chemotherapy. Therefore, early prediction of treatment responses is particularly important, which can avoid the additional costs of unnecessary toxic and ineffective treatment or overtreatment, and possibly increase the chances of receiving other potentially effective therapy. Over the past two decades, EGFR TKIs, e.g., erlotinib and gefitinib, have been proposed to be effective treatment strategies for NSCLC patients with EGFR mutations (9, 73). The mutation status of EGFR is an optimal predictor of treatment response to TKIs for patients with NSCLC (3, 4). Nevertheless, only a small subset of patients with EGFR mutations respond well to TKIs, especially erlotinib, which have prolonged survival (74, 75). The response rate of EGFR mutations to TKIs in patients with NSCLC varied greatly. Accordingly, new approaches are obviously needed to determine which patients will benefit from TKI treatment.

Traditionally, response evaluation for NSCLC patients harboring EGFR mutations treated with TKIs is usually based on anatomic imaging features that mainly present with static, and calculating the change of tumor size on CT and using Response Evaluation Criteria in Solid Tumors (RECIST) for classification (76, 77). However, the differences between atelectasis or fibrosis and residual neoplasm cannot be distinguished significantly by conventional anatomic imaging modalities (78, 79). Accordingly, the detection of early treatment response using these anatomic imaging tools has limited value. ^18^F-FDG PET/CT, a molecular and functional imaging method, has emerged as a powerful ability in diagnosing, staging, and evaluating outcomes for patients with NSCLC (80). In addition, ^18^F-FDG PET/CT has been proposed to be of great value in predicting the efficacy of radiotherapy, chemoradiotherapy, neoadjuvant chemotherapy, and combined intercalated chemotherapy and erlotinib in patients with advanced NSCLC (81–85).

As for patients with TKI-treated NSCLC, ^18^F-FDG PET/CT could be used to monitor response (**Table 2**) as early as 2 days after therapy, and those patients who had a partial metabolic response and stable metabolic disease would have a significantly longer PFS than those with progressive metabolic disease (86). Moreover, patients with partial remission and stable disease showed a decreasing uptake of ^18^F-FDG, while patients with progressive disease presented an increasing ^18^F-FDG uptake, which was the early response on day 2 and week 4 after treatment with gefitinib (87). In reality, low SUV_max_ of the primary tumor on ^18^F-FDG PET/CT scan usually correlated with a higher response rate than high SUV_max_ (88). The subsequent tumor reduction could be predicted by the decreasing uptake of ^18^F-FDG on PET/CT scan as an early response to the initiation of TKI treatment for patients harboring EGFR-mutated NSCLC (89). The histopathologic response could also be monitored by ^18^F-FDG PET/CT using SUV_max_ changes, and it had an advantage over traditional CT to evaluate histopathologic response for patients with neoadjuvant erlotinib-treated NSCLC (90–92).

**Table 2:** The findings of ^18^F-FDG PET/CT in evaluating treatment response and outcome for TKIs treated patients with NSCLC.

Studies	No. of patients		Clinical stage		Treatment strategies		Response evaluation time			Response rate				Prognosis (M)		Findings
	M	F	I–II	III–IV	Erlotinib	Gefitinib	Early	Interim	Late	CR	PR	PD	SD	PFS	OS	
Tiseo et al. (86)	35	18	0	53	√	–	D2	–	–	0	38%	15%	47%	2.1	7.6	Patients with early PMR and SMD have longer PFS and OS than PMD patients
Sunaga et al. (87)	0	5	0	5	–	√	D2	Wk4	–	0	40%	20%	40%	9.0	13.4	SUV_max_ decreased in patients with PR and SD during treatment
Na et al. (88)	47	37	0	84	–	√	–	Every 4 weeks	–	0	50%	13.1%	36.9%	3.0	7.5	Low SUV of the primary tumor shows higher response rate and longer PFS and OS
Koizumi et al. (89)	4	6	–	–	–	√	D7	–	–	0	100%	–	–	15.0	70% 1 year	Early reduction of SUV_max_ after therapy can predict subsequent tumor reduction
Aukema et al. (90)	8	15	21	2	√	–	D7	–	–	0	26%	4%	70%	–	–	^18^F-FDG PET/CT can predict early response to erlotinib treatment in patients with NSCLC
van Gool et al. (91)	18	25	37	6	√	–	D4–7	Wk3	–	0	33%	14%	53%	–	–	^18^F-FDG PET/CT can monitor early histopathologic response
van Gool et al. (92)	22	31	47	6	√	–	–	Wk3	–	0	15%	11%	60%	–	–	^18^F-FDG PET/CT has an advantage over CT to identify histopathologic response
Winther et al. (93)	28	22	0	50	√	–	D7	–	–	0	12%	14%	74%	2.7	6.0	Early increase in TLG correlates with radiological progression and shorter PFS and OS
Kahraman et al. (94)	13	17	0	30	√	–	D7	–	Wk6	NS	NS	NS	NS	NS	NS	Early ^18^F-FDG PET can monitor response and predict PFS
Cook et al. (95)	18	29	0	47	√	–	–	–	Wk6	34.4%		65.6%		–	14.1	Response to erlotinib is associated with reduced heterogeneity at ^18^F-FDG PET

CR, complete response; PR, partial response; SD, stable disease; PD, progressive disease; M, average months; D, day; Wk, week; PFS, progression-free survival; OS, overall survival; PMR, partial metabolic response; SMD, stable metabolic disease; PMD, progressive metabolic disease; SUV, standard uptake value; NS, not specified; “-”, not done.

The ^18^F-FDG metabolic activity of tumor on PET/CT scan can be revealed by several semiquantitative methods, e.g., SUV_max_, SUV_2Dpeak_ (2D peak SUV), SUV_3Dpeak_ (3D peak SUV), SUV_A50_ (3D isocontour at 50% of the maximum pixel value adapted for background), SUV_A41_ (3D isocontour at 41% of the maximum pixel value adapted for background), SUV_50_ (3D isocontour at 50% of the maximum pixel value), MTV, and TLG; these parameters have been demonstrated to be useful in monitoring response for patients with TKI-treated NSCLC (93, 94). However, the best parameters for the early response monitoring might be the SUV_max_, SUV_50_, SUV_A50_, and SUV_A41_ measured with ^18^F-FDG on PET/CT scan (94). Recently, tumor heterogeneity on ^18^F-FDG PET/CT has been evaluated for monitoring response in patients with erlotinib-treated NSCLC (95). The treatment response to erlotinib was related to the reduced heterogeneity of ^18^F-FDG PET. The change of first-order entropy was independently associated with treatment response and outcome (95). This study of NSCLC heterogeneity on ^18^F-FDG PET/CT opens a new window for monitoring therapy response.

As stated above, both EGFR and ^18^F-FDG PET/CT have potential value in monitoring TKI treatment response for NSCLC patients. Patients with mutant EGFR treated with TKIs benefit more than those with wild-type EGFR. ^18^F-FDG PET/CT demonstrates a high advantage in evaluating early treatment response. Several semiquantitative parameters of ^18^F-FDG metabolic activity present a significant role in assessing anatomical and histopathological responses for patients with NSCLC treated with TKIs. The heterogeneity of uptake of ^18^F-FDG on PET/CT may be a useful method to evaluate treatment response and prognosis for patients with NSCLC.

### Predicting Prognosis for Patients With Non-Small Cell Lung Cancer

The prognosis of patients with NSCLC is heterogeneous and varies greatly. Tumor-node-metastasis (TNM) classification is a measure to specify the disease extent for patients with NSCLC and plays a vital role in choosing a treatment strategy (96). ^18^F-FDG PET/CT has been demonstrated to be powerful in staging procedures and is more accurate than conventional CT in mediastinal staging for patients with NSCLC (97). Patients with advanced stage are usually incurable with a short life expectancy. Accordingly, the choice of treatment methods must be discreetly balanced between the potential benefits and ineffective side, effects and a precise evaluation of the prognosis of patients with NSCLC is of great importance.

In the past two decades, EGFR is a well-known predictive marker of outcome for patients with NSCLC who were treated with TKIs (98). TKIs have become the first-line treatment strategy in standard therapy for advanced-stage NSCLC harboring EGFR mutations, e.g., deletion of exon 19 or exon 21 or the L858R point mutations (7, 8). The mutation in exon 19 of EGFR was a reliable predictor of favorable survival for patients with NSCLC (55). Patients with activated EGFR mutations treated with TKIs had a higher response rate and longer PFS than those treated with standard cytotoxic chemotherapy (6). However, resistance inevitably develops eventually for patients with NSCLC who are treated with EGFR TKIs, and it is difficult for clinicians to predict the time of recurrence or progression owing to the wide range of PFS in individual patients. Some patients progressed several years after starting TKI therapy, while others progressed rapidly and spread widely after just a few months, usually with a median of 9–13 months (7–9). To our knowledge, there is currently no reliable clinical tool to predict the prognosis of EGFR mutant NSCLC patients treated with TKIs. Meanwhile, only two clinical features, TNM staging and performance status, have been considered to be significantly associated with prognosis in patients with NSCLC, but they need to be further validated by prospective studies (99).

The prognosis of patients with NSCLC from early stage to advanced stage has been evaluated by several studies with numerous procedures (100, 101). The role of ^18^F-FDG PET and high-resolution CT in predicting the prognosis for patients with clinical stage-IA NSCLC has been assessed, which showed that SUV_max_ of the primary tumor and ground-glass opacity ratios on high-resolution CT images were significant prognosticators of these patients, which should be kept in mind before selecting therapeutic strategies (101). In patients with advanced NSCLC treated with erlotinib, ^18^F-FDG PET presented a predictive effect as early as 1 week after initiation of treatment, predicting PFS, OS, and non-progression after 6 weeks of treatment, and was independent of EGFR mutational status (100). Several different semiquantitative parameters, e.g., SUV_max_, SUV_2Dpeak_, SUV_3Dpeak_, SUV_50_, SUV_A50_, and SUV_A41_, have been proved to be useful predictors of short-term prognosis in patients with advanced NSCLC in the early (1 week) and late (6 weeks) ^18^F-FDG PET/CT scans after initiation of erlotinib therapy (102). An updated systematic review and meta-analysis by the European lung cancer working party for the international association for the study of lung cancer staging project showed that SUV on ^18^F-FDG PET was potentially useful in predicting patient outcomes (13). Low SUVs of the primary tumor could predict favorable survival in NSCLC patients treated with TKIs (88, 102). Early evaluation of SUV_max_ changes on ^18^F-FDG PET at 2 days after initial treatment with gefitinib was of great significance to predict the clinical outcome of patients with lung adenocarcinoma (103). Moreover, early (day 14) partial metabolic response on ^18^F-FDG PET was independently associated with prolonged PFS and OS in patients with NSCLC treated with erlotinib (104).

In NSCLC patients with activating EGFR mutation, TLG has the potential role in predicting PFS and gefitinib resistance development on ^18^F-FDG PET (105). Measuring the baseline metabolic tumor burden with TLG before first-line TKIs will be very helpful to predict the time of acquired drug resistance (105). Intra-tumoral heterogeneity may be partially explained that not all patients with NSCLC harboring EGFR mutations will benefit from TKI therapy (106). Using an imaging tool may be a potentially simpler approach to assess tumor heterogeneity. Actually, heterogeneous textural parameters derived from baseline ^18^F-FDG PET/CT are demonstrated to be high predictors of clinical outcomes for NSCLC patients harboring EGFR mutations treated with TKIs (107). However, even though ^18^F-FDG PET/CT plays a vital role in predicting the prognosis for patients with NSCLC, contradictory results are also observed that SUV_max_ of the primary tumor cannot predict survival for patients with NSCLC (108, 109). Accordingly, furthermore, studies are needed to validate these findings to give clinicians an accurate recommendation.

## Conclusion

In summary, the ^18^F-FDG metabolic activity of NSCLC, as an extrinsic manifestation, plays a critical role in monitoring treatment response and evaluating prognosis. Several semiquantitative parameters (e.g., SUV_max_, MTV, and TLG) on ^18^F-FDG PET/CT can be used to reflect metabolic activity and tumor burden. EGFR mutation status, as an intrinsic factor, plays a vital role in guiding the implementation of treatment modalities (e.g., TKIs) and evaluating therapy efficacy and outcome for patients with NSCLC. Significant correlations are observed between ^18^F-FDG metabolic activity and EGFR mutation status, not only in biology but also in clinical practice. However, at present, there is still a lack of comprehensive evaluation of the association between ^18^F-FDG PET/CT and EGFR mutations in patients with NSCLC, e.g., using ^18^F-FDG PET/CT to predict EGFR mutation status and then monitor treatment response and evaluate the outcome, which needs to be carried out simultaneously in a large sample retrospective or prospective study.

## Author Contributions

MJ and JJZ were responsible for the conception of this review. All authors contributed to the article and approved the submitted version.

## Funding

This work was supported by the Medical Scientific Research Foundation of Zhejiang Province, China (Grant no. 2021KY1014), Research Foundation of Hwa Mei Hospital, University of Chinese Academy of Sciences, China (Grant no. 2022HMKY27), Ningbo Public Service Technology Foundation, China (Grant No. 2021S176), and Medical Science and Technology Project of Ningbo, China (Grant no. 2020Y10), Ningbo Clinical Medical Research Center of Imaging Medicine (Grant No. 2021L003), and Provincial and Municipal Co-construction Key Discipline of Medical Imaging (Grant No. 2022-S02).

## Conflict of Interest

The authors declare that the research was conducted in the absence of any commercial or financial relationships that could be construed as a potential conflict of interest.

## Publisher’s Note

All claims expressed in this article are solely those of the authors and do not necessarily represent those of their affiliated organizations, or those of the publisher, the editors and the reviewers. Any product that may be evaluated in this article, or claim that may be made by its manufacturer, is not guaranteed or endorsed by the publisher.
